# Exploring the mechanisms of artemisinin and its derivatives in the treatment of atopic dermatitis based on network pharmacology and molecular docking: A review

**DOI:** 10.1097/MD.0000000000042287

**Published:** 2025-05-09

**Authors:** Wenjing Xu, Qianyu Zhu, Jiaxing Chen, Junchen He, Aijie Yuan, Peng Cao, Litao Zhang

**Affiliations:** a Graduate School, Tianjin University of Traditional Chinese Medicine, Tianjin, China; b Graduate School, Tianjin Medical University, Tianjin, China; c Department of Dermatology, Tianjin Academy of Traditional Chinese Medicine Affiliated Hospital, Tianjin, China; d Tianjin Institute of Integrative Dermatology, Tianjin, China.

**Keywords:** artemisinin, artesunate, dihydroartemisinin, molecular docking, network pharmacology

## Abstract

This study investigates the therapeutic mechanisms of artemisinin (ARS) and its derivatives in atopic dermatitis (AD) using network pharmacology and molecular docking. Molecules and disease targets were screened using public databases, including SwissTargetPrediction, PharmMapper, and Genecards. Core targets were identified, and a protein-protein interaction (PPI) network was constructed using STRING and Cytoscape for topological analysis. Relevant data were obtained from the DAVID database for Gene Ontology (GO) and Kyoto Encyclopedia of Genes and Genomes (KEGG) enrichment analysis. Molecular docking of ARS and its derivatives with target genes was performed using AutoDock, with results visualized in Pymol. A functional PPI network was established, and molecular docking demonstrated strong binding activity between ARS derivatives and target protein. Mitogen-Activated Protein Kinase14 (MAPK14) and Mitogen-Activated Protein Kinase10 (MAPK10) was found to be a common target for their treatment of AD. ARS and its derivatives may treat AD by modulating pathways such as Prolactin signaling, cancer pathways, neuroactive ligand-receptor interaction, and IL-17 signaling. ARS and its derivatives have the potential to treat AD. Artemisinin, artesunate, dihydroartemisinin, artemether, artemisinin and artemisinone could potentially treat AD by targeting MAPK14 and MAPK10.

## 1. Introduction

Atopic dermatitis (AD) is a chronic, relapsing inflammatory skin disease with complex pathogenesis and diverse etiology.^[1]^ It is marked by persistent itching and eczema-like skin lesions clinically.^[2]^ Over the past few decades, the incidence of AD has been steadily increasing worldwide. In Europe, the prevalence of AD in adults is about 17.1%, while worldwide, the prevalence of AD in children is 15% to 20%.^[3,4]^ Persistent itching leads to sleep disturbances, mental health issues, and a cycle of scratching that worsens the condition, imposing significant psychological and social burdens.^[5]^ The pathophysiological mechanisms of AD are complex and multifactorial, involving genetic predisposition and hypersensitivity reactions in organs including the skin.^[6,7]^ Current treatments fail to fully cure AD due to its multifactorial nature.^[8]^

Current topical treatment strategies for AD involve the extensive use of emollients and anti-inflammatory therapies.^[9]^ The disruption of epidermal barrier function is considered a hallmark of AD, necessitating the use of at least 250 g of emollients per week for adolescents and adults to inhibit transepidermal water loss and reduce the need for anti-inflammatory medications.^[10]^ Topical treatment regimens also include anti-inflammatory therapy, with topical corticosteroids currently regarded as the first-line treatment for AD.^[11]^ In cases of mild AD, low- to mid-potency corticosteroids should be applied as needed. For moderate to severe AD, mid-potency corticosteroid regimens should be regularly combined with calcineurin inhibitors (such as tacrolimus or pimecrolimus) as part of the initial treatment strategy.^[2,12]^ While the use of emollients is entirely safe, topical corticosteroids may cause skin burning sensations and increase the risk of skin infections.^[13]^ Systemic treatment options for AD include immunosuppressants, JAK inhibitors and biologics. However, due to their significant side effects (such as liver and kidney function impairment, increased risk of infection, etc.) or high treatment costs, these therapies are reserved for acute exacerbations of AD to curb disease progression.^[14,15]^ The chronic, relapsing nature of AD, along with the side effects of long-term steroid use, presents a significant challenge in the management of this disease. Additionally, many patients experience flares after discontinuing treatment, highlighting the need for new therapeutic options that can provide lasting relief without long-term side effects.

ARS is a natural product extracted from the traditional Chinese plant Artemisia annua, and its derivatives include artesunate (ART), dihydroartemisinin (DHA), artemether (ARM), arteether (ARE), and artemisone (ARO).^[16]^ Existing research has proven that ARS and its derivatives have certain effects on inflammation and autoimmune diseases, providing a new therapeutic approach for inflammatory skin diseases with immune dysregulation, such as AD.^[17–20]^ Moreover, in recent years, research on artemisinin and its derivatives has increasingly expanded within the field of dermatology, encompassing studies on rosacea, psoriasis, and skin photoaging. The findings consistently demonstrate that artemisinin and its derivatives exhibit positive therapeutic effects in these conditions.^[21–23]^ Currently, research on the use of artemisinin for the treatment of AD remains in the experimental stage, primarily conducted in animal models. Bai et al demonstrated that artesunate significantly decreased mast cell counts and TNF-α levels in a DNCB-induced AD mouse model.^[24]^ Xue et al observed that treatment with dihydroartemisinin in DNCB-induced AD mice significantly reduced mast cell levels, with a positive correlation between the reduction and the administered dose of dihydroartemisinin.^[25]^ Therefore, we conclude that ARS and its derivatives may be effective in AD patients by reducing mast cell levels, moreover, in traditional Chinese medicine, artemisia annua has anti-inflammatory effects as a traditional Chinese medicine, which has also been confirmed in modern studies.^[26,27]^ As mentioned above, ARS and its derivatives has the potential and feasibility to effectively treat AD, however, the exact mechanism of action and which molecules are more effective in treating AD are still need to be explored.

Network pharmacology is a subject based on systems biology theory, biological system network analysis and the selection of specific signal nodes for multi-target drug molecule design, which provides a new research idea for exploring the relationship between drugs and diseases.^[28,29]^ Network pharmacology has been widely applied in the field of dermatology to explore the potential mechanisms by which drugs exert their effects in various skin diseases. For example, it has been utilized to investigate the multi-target interactions and signaling pathways of traditional Chinese medicine compounds in psoriasis, to identify the molecular networks involved in the anti-inflammatory and immunomodulatory effects of biologics in AD, and to elucidate the mechanisms of action of small-molecule inhibitors in melanoma.^[30,31]^ Based on this, this study uses network pharmacology to analyze the pharmacological mechanisms of ARS and its derivatives in treating AD, hoping to provide a reference for further experimental research.

## 2. Methods

### 2.1. Acquisition of basic information on ARS and its derivatives

The canonical SMILES of ARS and its derivatives were queried in PubChem (https://pubchem.ncbi.nlm.nih.gov/). SwissADME (https://www.swissadme.ch/) was used to preliminarily assess whether ARS and its derivatives have effective components. The potential targets of ARS and its derivatives were obtained from the SwissTargetPrediction database (https://old.swisstargetprediction.ch/) (Homo sapiens, probability > 0). Pubchem: organic small molecule bioactivity database, providing a wealth of chemical and physical properties, biological activity, safety and toxicity information. SwissADME: a database that provides a range of computational models for drugs, including solubility, plasma protein binding, liver metabolism, renal excretion, etc. SwissTargetPrediction: An online database for predicting targets of bioactive small molecules in humans and other vertebrates. The database contains 370,000 known compounds and more than 3000 targets.

### 2.2. Acquisition of disease-related targets

Atopic dermatitis-related targets were screened from the GeneCards (https://www.genecards.org/), Online Mendelian Inheritance in Man (OMIM) (https://omim.org/), and Therapeutic Target Database (TTD) (https://db.idrblab.net/ttd/) databases, duplicate values were removed to serve as potential therapeutic targets for AD. Genecards: A searchable gene database that consolidates resources from about 150 gene-focused databases, providing information on virtually all known human genes. OMIM: an integrated and authoritative database. It studies the relationship between human phenotypes and genotypes. OMIM contains information on all known Mendelian diseases. It also includes data on more than 16,000 genes. TTD: The first online database in the world to provide free information on drug targets globally.

### 2.3. Venn diagram analysis

The online tool Jvenn (https://jvenn.toulouse.inra.fr/app/index.html) was used to obtain overlapping targets between drugs and diseases. At the same time, Jvenn analysis was employed to identify the shared therapeutic targets among 6 drugs, elucidating the common potential targets of ARS and its derivatives for the treatment of AD.^[32]^

### 2.4. Construction of PPI network and screening of core targets

The potential therapeutic targets of each drug were input into the STRING database (https://cn.string-db.org/), with the protein type set to Homo sapiens and the required minimum interaction score set to medium confidence (0.400), which is a common threshold that strikes a balance between coverage and reliability.^[33]^ After hiding the disconnected nodes in the network, the PPI network diagrams for the 6 drugs were obtained. The results were imported into Cytoscape software, and the Centiscape plugin tool in the software was used to screen core targets based on Betweenness, Closeness, and Degree values to construct a new PPI network diagram.

### 2.5. GO and KEGG pathway enrichment analysis

After obtaining GO and KEGG-related data from the DAVID database, the data were processed using Excel, and a *P* value of < .05 was defined as significant enrichment. According to the size of the *P* value, the top 10 molecular functions (MF), cellular components (CC), and biological processes (BP) and the top 20 KEGG-enriched pathways were presented through bubble charts.

### 2.6. Molecular docking

The common targets identified through the intersection analysis of the 6 drugs with the disease were selected, along with the top-ranked core targets for molecular docking studies, to elucidate the therapeutic mechanisms of ARS and its derivatives in AD treatment. The 3D structure data of each molecule in sdf format was downloaded from PubChem, and the sdf format files were converted to pdb format using Open Babel (https://open-babel.readthedocs.io/en/latest/Installation/install.html). Suitable target proteins were screened from the Uniprot database (https://www.uniprot.org/) with the conditions of Reviewed and Human, and the data files of each target protein were downloaded from the PDB database based on the PDB ID. The ligand and receptor data were imported into the Autodock software for molecular docking, and the binding energy was recorded. Autodock is an open-source molecular simulation software. It’s primarily used for docking of small molecules to large biomolecules. The results of the docking were visualized using Pymol.

## 3. Results

### 3.1. Basic information on ARS and its derivatives

The canonical SMILES and basic 2D structure diagrams of ARS and its derivatives are shown in Table 1. The drug utilization information obtained from the SwissTargetPrediction is shown in Table 2. A total of 34 intersection targets for artemisinin, 50 for artesunate, 49 for dihydroartemisinin, 40 for arteether, 48 for artemisone, and 28 for artemether were ultimately obtained.

**Table 1:** Basic information on ARS and its derivatives.

Name	Canonical smiles
Artemisinin	CC1CCC2C(C(=O)OC3C24C1CCC(O3)(OO4)C)C
Artesunate	CC1CCC2C(C(OC3C24C1CCC(O3)(OO4)C)OC(=O)CCC(=O)O)C
Dihydroartemisinin	CC1CCC2C(C(OC3C24C1CCC(O3)(OO4)C)O)C
Artemether	CC1CCC2C(C(OC3C24C1CCC(O3)(OO4)C)OC)C
Arteether	CCOC1C(C2CCC(C3C24C(O1)OC(CC3)(OO4)C)C)C
Artemisone	CC1CCC2C(C(OC3C24C1CCC(O3)(OO4)C)N5CCS(=O)(=O)CC5)C

ARS = artemisinin.

**Table 2:** Drug utilization of ARS and its derivatives.

Name	Gatrointestinal absorption	Lipinski (Pfizer) filter	Ghose filter	Veber (GSK) filter	Egan (Pharmacia) filter	Muegge (Bayer) filter	Abbott (Bioavailability) Score
Artemisinin	High	Yes	Yes	Yes	Yes	Yes	0.55
Artesunate	High	Yes	Yes	Yes	Yes	Yes	0.56
Dihydroartemisinin	High	Yes	Yes	Yes	Yes	Yes	0.55
Artemether	High	Yes	Yes	Yes	Yes	Yes	0.55
Arteether	High	Yes	Yes	Yes	Yes	Yes	0.55
Artemisone	High	Yes	Yes	Yes	Yes	Yes	0.55

ARS = artemisinin.

### 3.2. Common targets of ARS and its derivatives with AD

**Figure 1.**
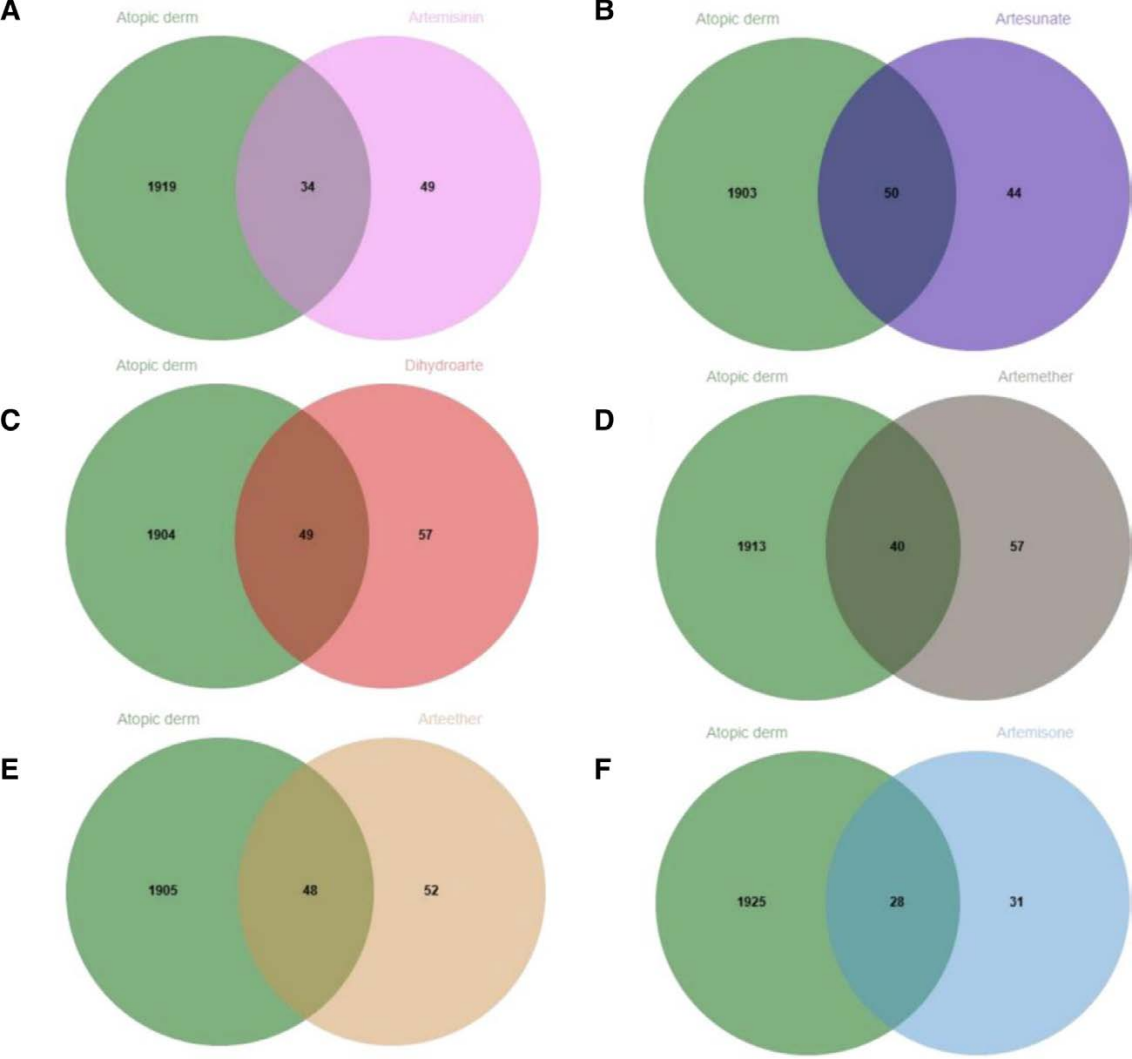
(A) The Venn diagram of targets for treating AD-ARS. (B) The Venn diagram of targets for treating AD-ART. (C) The Venn diagram of targets for treating AD-DHA. (D) The Venn diagram of targets for treating AD-ARM. (E) The Venn diagram of targets for treating AD-ARE. (F) The Venn diagram of targets for treating AD-ARO. AD = atopic dermatitis, ARE = arteether, ARM = artemether, ARO = artemisone, ARS = artemisinin, ART = artesunate, DHA = dihydroartemisinin.

**Figure 2.**
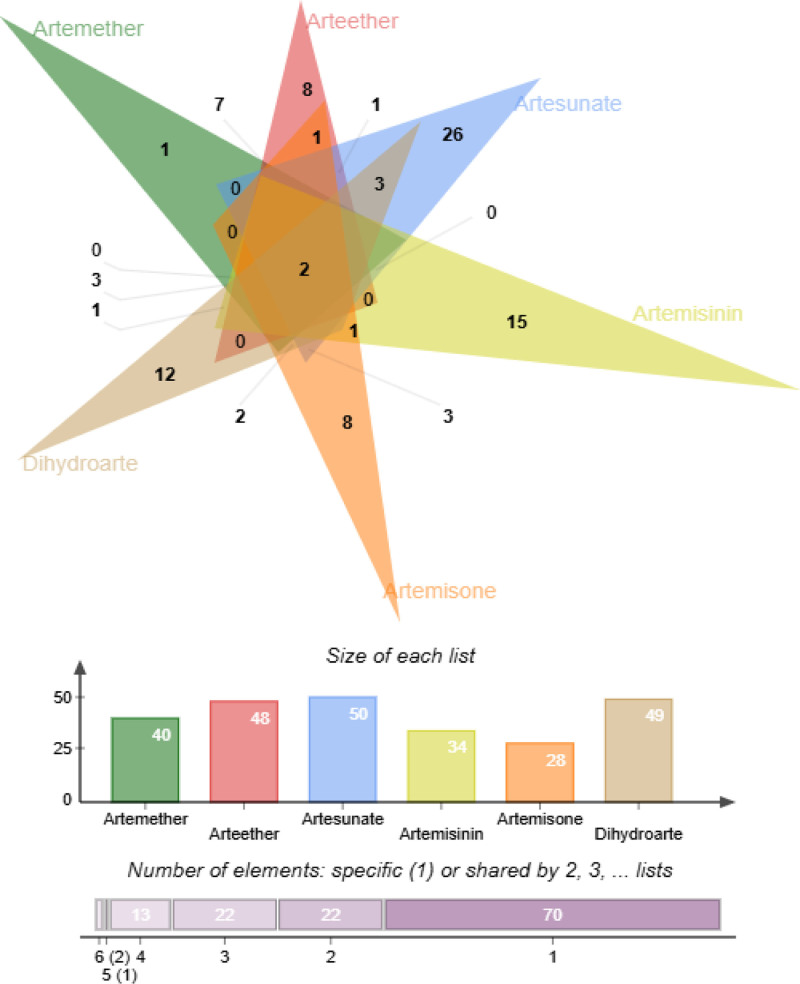
Common intersection targets of artemisinin and its derivatives in the treatment of AD. AD = atopic dermatitis.

Using “Atopic dermatitis” as the keyword, core targets were screened from the GeneCards, TTD, and OMIM databases, resulting in 1953 unique disease targets after de-duplication. Venn diagrams are shown in Figure 1A–F. A Venn diagram illustrating the common targets effective for AD across the 6 drugs is presented in Figure 2. Ultimately, ARS, ART, DHA, ARM, ARE, and ARO were found to have 34, 50, 49, 40, 48, and 28 intersecting targets with AD, respectively. The two common targets for the treatment of AD by the 6 drugs are Mitogen-Activated Protein Kinase 14 (MAPK14) and Mitogen-Activated Protein Kinase 10 (MAPK10), with specific information provided in Table 3.

**Table 3:** Common intersection targets of artemisinin and its derivatives in the treatment of AD.

Artemether Arteether	HRH4, SYK, STAT5B, CTSS, HRH2, NTRK1, HRH3
Arteether Artesunate	CASP3
Arteether Artemisone	MMP13
Artesunate Artemisone	ELANE, NR3C1, PPARG
Artemisinin Artemisone	PDGFRB
Artemether Arteether Artemisinin	HTR2A, ADORA1, ADORA2A, CYP19A1
Artemether Arteether Artemisone	TLR9
Arteether Artemisinin Artemisone	HSD11B1
Artemether Arteether Artesunate Artemisinin	ESR2
Artemether Arteether Artemisinin Artemisone	HMOX1, MAPK8
Arteether Artesunate Artemisinin Artemisone	GSK3B
Artemisone Dihydroarte	PSENEN, NCSTN
Artemisinin Dihydroarte	EGFR
Artemisinin Artemisone Dihydroarte	MAP2K1
Artesunate Dihydroarte	MAPK1, HMGCR, TYMS
Artesunate Artemisone Dihydroarte	MMP9, MMP1, MMP8
Arteether Artesunate Dihydroarte	PIK3CA, CASP8
Arteether Artesunate Artemisinin Dihydroarte	CYP1A2
Artemether Dihydroarte	JAK1, ABCC9, CA2
Artemether Artesunate Dihydroarte	EDNRA, EDNRB, CASP7
Artemether Arteether Dihydroarte	PTAFR, JAK2, OPRK1, ALOX12, CHRM3, PARP1, TERT
Artemether Arteether Artemisone Dihydroarte	PTGS2
Artemether Arteether Artemisinin Dihydroarte	JAK3, SLC6A4, AR, CHRM2
Artemether Arteether Artesunate Dihydroarte	CASP1, OPRM1, ADORA2B
Artemether Arteether Artesunate Artemisone Dihydroarte	MMP2
Artemether Arteether Artesunate Artemisinin Artemisone Dihydroarte	MAPK14, MAPK10

AD = atopic dermatitis, MAPK = Mitogen-Activated Protein Kinase.

### 3.3. PPI network analysis and core target identification

**Figure 3.**
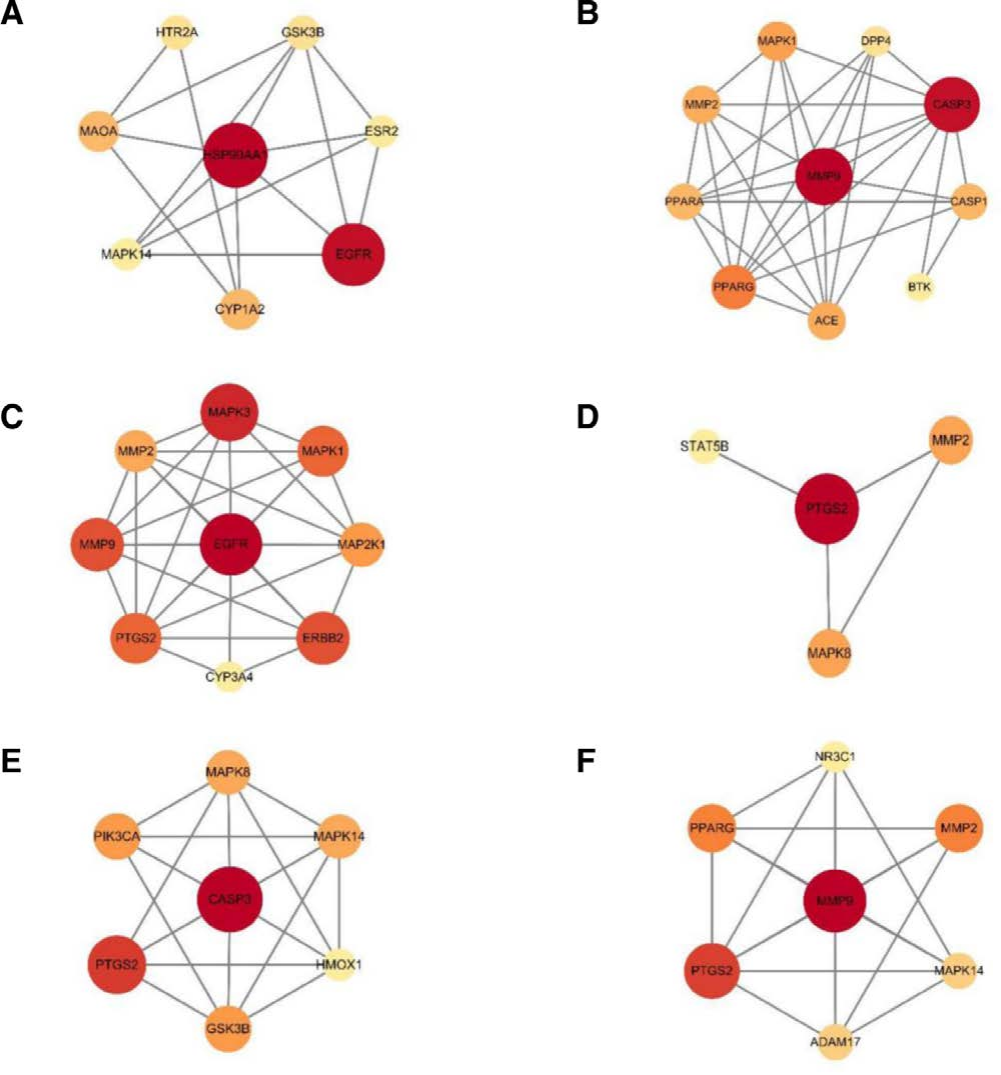
(A) The PPI network of ARS. (B) The PPI network of ART. (C) The PPI network of DHA. (D) The PPI network of ARM. (E) The PPI network of ARE. (F) The PPI network of ARO. ARE = arteether, ARM = artemether, ARO = artemisone, ARS = artemisinin, ART = artesunate, DHA = dihydroartemisinin, PPI = protein-protein interaction.

Protein-Protein Interaction (PPI) network diagrams illustrate the interactions between proteins. Using Cytoscape software, the analysis, selection, and construction of new core target network diagrams were performed. The results indicated that the first core target for ARS is Heat Shock Protein 90 Alpha Family Class A Member 1 (HSP90AA1), for ART is Matrix Metalloproteinase 9 (MMP9), for DHA is Epidermal Growth Factor Recepto (EGFR), for ARM is Prostaglandin-endoperoxide synthase 2 (PTGS2), for ARE is Caspase 3 (CASP3), and forARO is MMP9. The PPI network diagram for each molecule is shown in Figure 3A–F.

### 3.4. GO enrichment analysis

**Figure 4.**
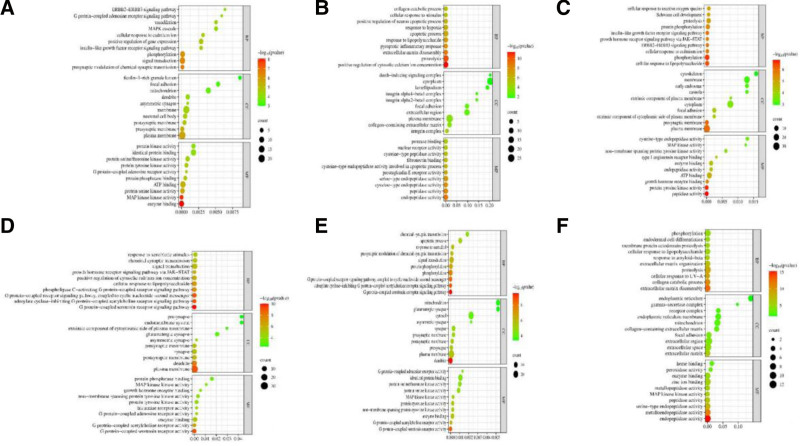
(A) GO enrichment about AD-ARS. (B) GO enrichment about AD-ART. (C) GO enrichment about AD-DHA. (D) GO enrichment about AD-ARM. (E) GO enrichment about AD-ARE. (F) GO enrichment about AD-ARO. AD = atopic dermatitis, ARE = arteether, ARM = artemether, ARO = artemisone, ARS = artemisinin, ART = artesunate, DHA = dihydroartemisinin, GO = Gene Ontology.

Conduct GO enrichment analysis for artemisinin and its 5 derivatives, and visualize the results. Bubble charts are used to describe the BP, CC, and MF of each molecule in treating AD. The Y-axis of the bubble chart represents the names, the X-axis represents the False Discovery Rate (FDR), the color represents the significance of the *P*-value, and the size of the bubbles represents the number of genes. Results can be seen in the Figure 4. The biological processes mainly involved in ARS treatment of AD include presynaptic modulation of chemical synaptic transmission, signal transduction, etc., cellular components mainly involve plasma membrane, presynaptic membrane, etc., and molecular functions mainly involve enzyme binding, MAP kinase kinase activity, etc.; The biological processes mainly involved in ART treatment of AD include positive regulation of cytosolic calcium ion concentration, proteolysis, etc., cellular components mainly involve collagen-containing extracellular matrix, plasma membrane, etc., and molecular functions mainly involve endopeptidase activity, peptidase activity, etc.; The biological processes mainly involved in DHA treatment of AD include cellular response to lipopolysaccharide, phosphorylation, etc., cellular components mainly involve plasma membrane, presynaptic membrane, etc., and molecular functions mainly involve peptidase activity, protein tyrosine kinase activity, etc.; The biological processes mainly involved in ARM treatment of AD include G protein-coupled serotonin receptor signaling pathway, adenylate cyclase-inhibiting G protein-coupled acetylcholine receptor signaling pathway, etc., cellular components mainly involve plasma membrane, dendrite, etc., and molecular functions mainly involve G protein-coupled serotonin receptor activity, G protein-coupled acetylcholine receptor activity, etc.; The biological processes mainly involved in ARE treatment of AD include G protein-coupled serotonin receptor signaling pathway, adenylate cyclase-inhibiting G protein-coupled acetylcholine receptor signaling pathway, etc., cellular components mainly involve dendrite, presynapse, etc., and molecular functions mainly involve G protein-coupled serotonin receptor activity, G protein-coupled acetylcholine receptor activity, enzyme binding, etc.; The biological processes mainly involved in ARO treatment of AD include extracellular matrix disassembly, collagen catabolic process, etc., cellular components mainly involve extracellular matrix, extracellular space, etc., and molecular functions mainly involve endopeptidase activity, metallopeptidase activity, etc. Intersection processing of BP, CC, and MF for each molecule revealed 12 common BPs, 2 shared CCs, and 4 shared MFs, suggesting that artemisinin and its derivatives may exert their therapeutic and preventive effects on AD through these biological functions. Detailed information can be found in Table 4.

**Table 4:** The common GO terms of 6 molecules.

GO	Common
BP	Signal transduction, phosphorylation, positive regulation of gene expression, cellular response to cadmium ion, positive regulation of smooth muscle cell proliferation, protein phosphorylation, cellular response to stress, response to xenobiotic stimulus, intracellular signal transduction, stress-activated MAPK cascade, cellular response to lipopolysaccharide, angiogenesis
CC	Plasma membrane, mitochondrion
MF	Enzyme binding, MAP kinase activity, steroid binding, protein binding

BP = biological process, CC = cellular component, GO = gene ontology, MAPK = Mitogen-Activated Protein Kinase, MF = molecular function.

### 3.5. KEGG enrichment analysis

**Figure 5.**
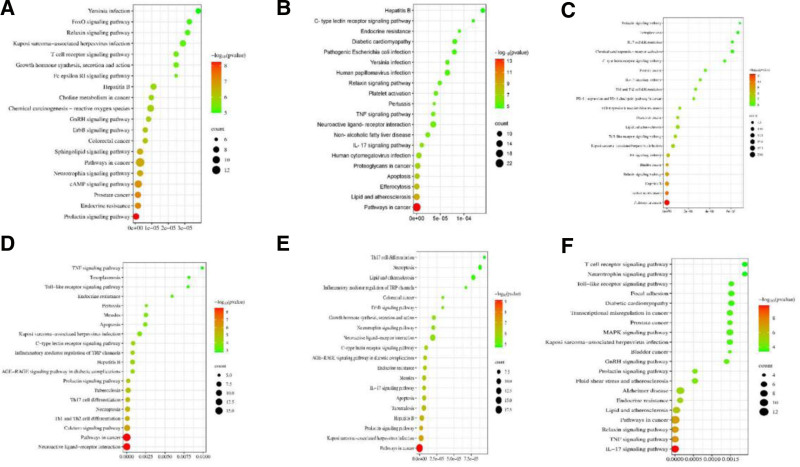
(A) KEGG enrichment about AD-ARS. (B) KEGG enrichment about AD-ART. (C) KEGG enrichment about AD-DHA. (D) KEGG enrichment about AD-ARM. (E) KEGG enrichment about AD-ARE. (F) KEGG enrichment about AD-ARO. AD = atopic dermatitis, ARE = arteether, ARM = artemether, ARO = artemisone, ARS = artemisinin, ART = artesunate, DHA = dihydroartemisinin, KEGG = Kyoto Encyclopedia of Genes and Genomes.

The KEGG enrichment analysis results indicate that the treatment of AD with ARS involves signaling pathways such as the Prolactin signaling pathway and Endocrine resistance. For ART in the treatment of AD, pathways involved include Pathways in cancer and Lipid and atherosclerosis. DHA’s treatment of AD is associated with Pathways in cancer and Endocrine resistance. ARM’s involvement in treating AD encompasses the Neuroactive ligand-receptor interaction and Pathways in cancer. ARE’s treatment of AD is linked to Pathways in cancer and Kaposi sarcoma-associated herpesvirus infection. Finally, ARO’s treatment of AD involves the Interleukin-17 (IL-17) signaling pathway and Tumor Necrosis Factor (TNF) signaling pathway. The results are shown in Figure 5.

### 3.6. Molecular docking

**Figure 6.**
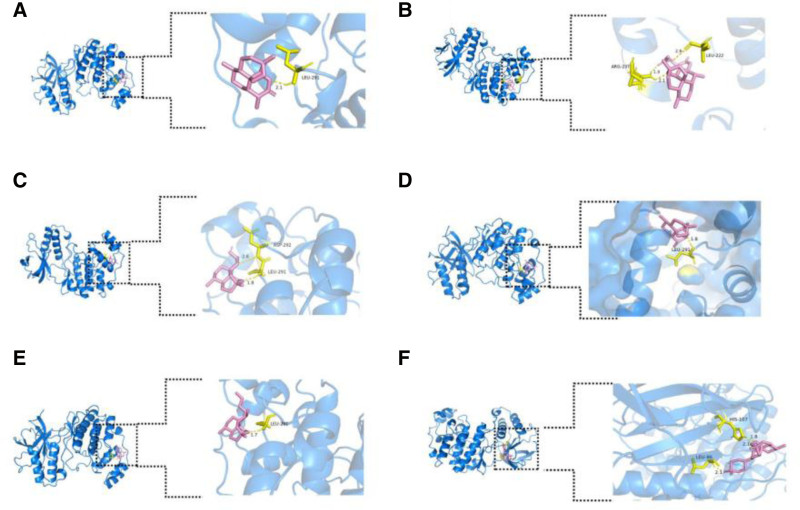
(A) Visualization of MAPK14 docking with ARS. (B) Visualization of MAPK14 docking with ART. (C) Visualization of MAPK14 docking with DHA. (D) Visualization of MAPK14 docking with ARM. (E) Visualization of MAPK14 docking with ARE. (F) Visualization of MAPK14 docking with ARO. AD = atopic dermatitis, ARE = arteether, ARM = artemether, ARO = artemisone, ARS = artemisinin, ART = artesunate, DHA = dihydroartemisinin, MAPK = Mitogen-Activated Protein Kinase.

**Figure 7.**
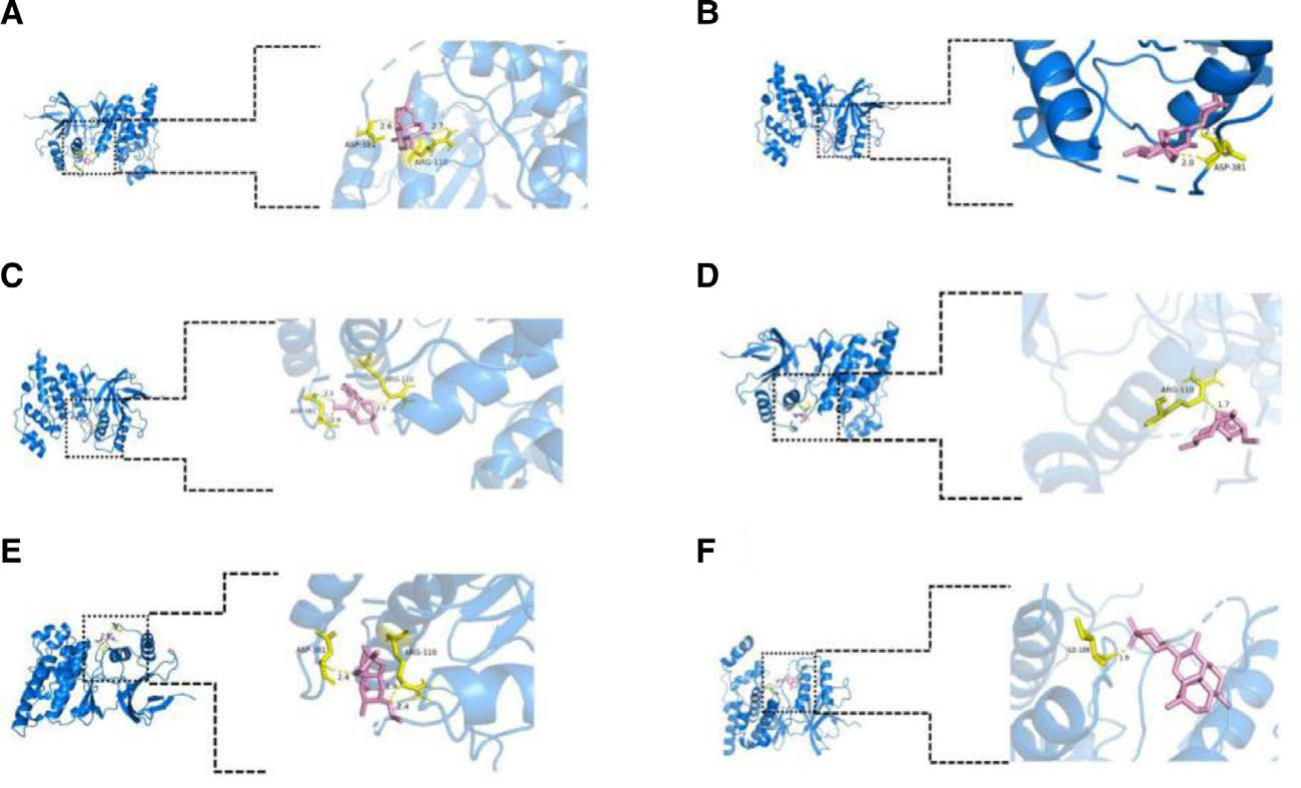
(A) Visualization of MAPK10 docking with ARS. (B) Visualization of MAPK10 docking with ART. (C) Visualization of MAPK10 docking with DHA. (D) Visualization of MAPK10 docking with ARM. (E) Visualization of MAPK10 docking with ARE. (F) Visualization of MAPK10 docking with ARO. AD = atopic dermatitis, ARE = arteether, ARM = artemether, ARO = artemisone, ARS = artemisinin, ART = artesunate, DHA = dihydroartemisinin, MAPK = Mitogen-Activated Protein Kinase.

Molecular docking was performed for the 6 molecules with their respective primary targets for the treatment of AD, followed by docking with the common targets MAPK14 and MAPK10. It is generally accepted that the smaller the binding energy, the stronger the binding interaction between the ligand and the receptor. A binding energy of less than −4.25 kcal/mol indicates a certain level of binding between the ligand and the receptor, while a binding energy of less than −5.0 kcal/mol suggests good binding activity. Furthermore, a binding affinity of less than −7.0 kcal/mol signifies strong binding activity. In this molecular docking study, the binding energies between the target proteins and the active compounds ranged from −5.67 to −8.40 kcal/mol. Molecular docking confirmed strong binding between the 6 compounds and their primary and shared targets. The visualization of the docking of each molecule with a common target is shown in the Figures 6 and 7, the results of molecular docking are shown in Table 5.

**Table 5:** The result of docking.

Name	Target	UNIPROT ID	PDB ID	Molecular binding energy (Kcal/mol)
artemisinin	MAPK14	Q16539	1A9U	−7.05
artesunate	MAPK14	Q16539	1A9U	−6.22
dihydroartemisinin	MAPK14	Q16539	1A9U	−6.91
artemether	MAPK14	Q16539	1A9U	−7.44
arteether	MAPK14	Q16539	1A9U	−7.76
artemisinone	MAPK14	Q16539	1A9U	−8.40
artemisinin	MAPK10	P53779	1PMN	−6.84
artesunate	MAPK10	P53779	1PMN	−5.67
dihydroartemisinin	MAPK10	P53779	1PMN	−7.00
artemether	MAPK10	P53779	1PMN	−6.57
arteether	MAPK10	P53779	1PMN	−6.45
artemisinone	MAPK10	P53779	1PMN	−8.23
artemisinin	HSP90AA1	P07900	1OSF	−7.41
artesunate	MMP9	P14780	1ITV	−5.89
dihydroartemisinin	EGFR	P00533	1XKK	−6.83
artemether	PTGS2	P35354	5F19	−6.78
arteether	CASP3	P42574	1CP3	−6.23
artemisinone	MMP9	P14780	1ITV	−7.90

CASP3 = Caspase 3, EGFR = Epidermal Growth Factor Recepto, HSP90AA1 = Heat Shock Protein 90 Alpha Family Class A Member 1, MAPK = Mitogen-Activated Protein Kinase, MMP9 = Matrix Metalloproteinase 9, PTGS2 = Prostaglandin-endoperoxide synthase 2.

## 4. Discussion

AD is a highly heterogeneous inflammatory skin disease with complex pathophysiological mechanisms, the core of its immunological mechanism lying in the imbalance of Th1/Th2 cells, which triggers a series of complex inflammatory responses.^[6,34]^ Langerhans cells and inflammatory dendritic cells in the skin recognize and present allergens, activating Th2 cells to secrete cytokines such as IL-4, IL-5, and IL-13.^[35,36]^ These factors further stimulate inflammatory cells, leading to localized skin inflammation. Meanwhile, the activation of Th1 cell responses increases the release of inflammatory mediators like IL-1 and TNF-α, exacerbating the skin’s inflammatory process.^[37]^ The interplay between these immune cells and the regulation of the cytokine network are key elements in the pathophysiology of AD.^[29,38]^ Current treatment approaches for AD include oral antihistamines, immunosuppressants, and topical corticosteroids.^[11,39,40]^ In recent years, biologic agents and small molecule targeted drugs have also become frontline therapies for AD.^[9,41]^

Artemisinin, a natural plant extract, has demonstrated diverse therapeutic effects beyond its antimalarial properties.^[42–45]^ Moreover, the research of topical artemisinin and its derivatives in skin diseases has also made some progress. Network pharmacology can predict the mechanisms of drug action in diseases by establishing molecular interaction networks and signal transduction models.^[29,46]^ This study employed network pharmacology to predict the therapeutic targets and mechanisms of ARS and its derivatives in AD.

Building on this foundation, we employed an innovative approach integrating network pharmacology and molecular docking to systematically explore the therapeutic potential of artemisinin and its 5 derivatives in the context of AD. Our findings revealed that all 6 compounds exhibit promising anti-AD properties, with MAPK10 and MAPK14 identified as shared molecular targets. Furthermore, each compound demonstrated unique key targets associated with AD pathogenesis. Molecular docking analysis confirmed strong binding affinities between the 6 molecules and both the common targets and their respective primary targets. These results collectively suggest that artemisinin and its derivatives possess substantial therapeutic potential for the treatment of AD.

This research has found that MMP9 is the primary core target for the treatment of AD with artesunate and artemether, while HSP90AA1, EGFR, PTGS2, and CASP3 are the most significant targets for the treatment of AD with artemisinin, dihydroartemisinin, artemether, and arteether, respectively. MAPK14 and MAPK10 is a common target for their treatment of AD. Mitogen-activated protein kinases (MAPKs) are a family of crucial signaling molecules involved in regulating various biological processes, including cell proliferation, differentiation, inflammatory responses, and immune reactions.^[47]^ In the pathogenesis of AD, the MAPK signaling pathway plays a significant role by modulating immune cell activation, the release of inflammatory cytokines, and epidermal barrier function.^[48]^ Specifically, MAPK10 contributes to AD primarily by activating the AP-1 transcription factor, upregulating the expression of pro-inflammatory cytokines such as IL-6 and TNF-α, thereby exacerbating skin inflammation. Additionally, abnormal activation of MAPK10 may increase keratinocyte apoptosis, further impairing epidermal barrier function and aggravating skin dryness and water loss in AD.^[49]^ On the other hand, MAPK14 promotes chronic inflammatory states by regulating Th2-type immune responses and enhancing the release of inflammatory mediators such as IL-4 and IL-13.^[50]^ Studies have shown that the activation of MAPK14 is also associated with the transmission of itch signals, potentially exacerbating AD symptoms through the modulation of neuroimmune interactions.^[51]^ The synergistic effects of MAPK10 and MAPK14 collectively drive the pathological progression of AD, providing a potential theoretical basis for therapeutic strategies targeting the MAPK signaling pathway. Studies have shown that MMP9 is a protease-activated enzyme that degrades the extracellular matrix. It can also activate mast cells and promote the release of allergic mediators, participating in the regulation of allergies. Therefore, the overexpression of MMP9 in the skin of AD patients promotes the infiltration of skin inflammatory cells and exacerbates the inflammatory response.^[52,53]^ HSP90AA1 is a heat shock protein involved in protein folding and stability. One Studie suggests that HSP90AA1, as a glucocorticoid receptor chaperone, can affect the efficacy of glucocorticoid therapy in lupus erythematosus, potentially playing a similar role in AD patients.^[54]^ Additionally, inhibition of HSP90AA1 has been found to alleviate inflammation in AD models, which indicating that HSP90AA1 inhibition may offer a novel therapeutic mechanism for AD.^[46,55]^ EGFR, a crucial epidermal growth factor receptor, stimulates epidermal cell proliferation and inhibits their differentiation. In AD, its overactivation can promote skin cell hyperplasia and inflammatory responses. Thus, aberrant EGFR signaling may be associated with impaired skin barrier function and the maintenance of chronic inflammation in AD patients.^[56,57]^ PTGS2, also known as COX-2, is an enzyme that promotes the synthesis of prostaglandins, which can exacerbate skin inflammation. In patients with AD, the overactivation of PTGS2 can intensify the “itch-scratch” cycle, leading to a worsening of AD symptoms.^[58]^ CASP3 is a protease that performs apoptosis and plays an important role in cell differentiation, apoptosis and inflammation. The apoptosis of keratinocytes and cellular inflammation play an important role in the pathogenesis of AD. Therefore, it is inferred that abnormal expression of CASP3 may participate in the pathogenesis of AD through the destruction of skin barrier function and the imbalance of apoptosis regulation of immune cells.^[59,60]^

KEGG analysis results show that the targets of the 6 molecules in the treatment of AD are significantly enriched in the Prolactin signaling pathway, Pathways in cancer, neuroactive ligand-receptor interaction, and L-17 signaling pathway. Prolactin, a pleiotropic hormone, activates signal pathways through its specific receptor, affecting cell proliferation, differentiation, and apoptosis.^[61]^ In AD, abnormalities in the Prolactin signaling pathway may be associated with skin barrier dysfunction, immune cell dysfunction, and the abnormal production of inflammatory mediators. For instance, the sebaceous gland is a crucial site for adult hormone formation, and sebocytes express a broad spectrum of hormone receptors. A study has suggested that, similar to androgens, prolactin also influences sebaceous gland function, thereby playing a role in the pathogenesis of AD.^[62]^ Cancer-related pathways, such as Wnt/β-catenin, NF-κB, and PI3K/AKT, although mainly related to the occurrence and development of tumors, have been found in recent years to also play an important role in the pathogenesis of AD. The abnormal activation of these pathways may lead to skin barrier dysfunction, abnormal activation of immune cells, and excessive production of inflammatory mediators, thus participating in the pathogenesis of AD.^[63–65]^ For instance, the NF-κB pathway plays a critical role in both cancer and AD. This pathway regulates the expression of various inflammatory mediators, such as cytokines and chemokines, which are crucial in the inflammatory response of AD and the tumor microenvironment of cancer.^[66,67]^ Additionally, the JAK-STAT signaling pathway is significantly involved in both cancer and AD. In AD, this pathway mediates Th2-type immune responses, while in cancer, it contributes to tumor cell proliferation and survival.^[68]^ Neuroactive ligand-receptor interactions are an important part of the neuroimmune system, participating in the regulation of immune responses and inflammatory processes. In AD, abnormal interactions between neuroactive ligands (such as nerve growth factor, vasoactive intestinal polypeptide, etc.) and their receptors may be associated with the abnormal activation of immune cells and the excessive production of inflammatory mediators.^[69,70]^ In AD, the abnormal activation of the L-17 signaling pathway may lead to skin barrier dysfunction, abnormal activation of immune cells, and excessive production of inflammatory mediators.^[71]^ IL-17 is involved in the pathogenesis of AD by inducing the production of inflammatory mediators and abnormal proliferation of skin cells.^[72]^Moreover, study suggests that IL-17, a crucial pro-inflammatory cytokine, is primarily secreted by CD4 + helper T cells and subsets of innate lymphoid cells. IL-17A has been implicated in the pathogenesis of inflammatory diseases, such as psoriasis and AD.^[73]^

This study utilized online databases and computational predictions to identify effective components and targets, constructed a protein-protein interaction (PPI) network, analyzed key target genes, and performed GO and KEGG analyses to explore the pharmacological mechanisms of artemisinin in treating AD. Anti-inflammatory and antipruritic effects are the primary therapeutic principles for AD, making corticosteroids a standard clinical treatment for this condition.^[74]^ As traditional Chinese medicinal extracts, artemisinin and its derivatives have been well-documented for their anti-inflammatory properties.^[75]^ Among them, ART and DHA have been investigated in animal models of AD. Experimental studies on ART have demonstrated its ability to alleviate DNCB-induced AD by inhibiting the release of inflammatory cytokines and downregulating Th17 cell responses in mice.^[24]^ Similarly, another study found that DHA may modulate local immunity by suppressing mast cell infiltration in lesions, thereby improving AD symptoms. These findings align with the results of the present study, which suggest that artemisinin and its derivatives hold therapeutic potential for AD.^[25]^ However, the precise mechanisms underlying their efficacy warrant further investigation. The results of this study provide a basis for our subsequent animal experiments, which we anticipate will validate this research. Additionally, the study offers theoretical support for future research on synthetic drugs for AD.

## 5. Conclusion

This study demonstrates that ARS and its derivatives—ART, DHA, ARM, ARE, and ARO—exhibit therapeutic potential for AD by targeting shared molecular pathways, particularly MAPK14 and MAPK10. Molecular docking confirmed strong binding affinities, supporting their anti-inflammatory and immunomodulatory effects. These findings highlight ARS and its derivatives as promising multi-target agents for AD treatment, offering a foundation for further experimental validation.

The authors kindly thank Mingjie Chen (Shanghai NewCore Biotechnology Co., Ltd.) for providing data analysis and visualization support.

## Author contributions

**Conceptualization:** Wenjing Xu.

**Data curation:** Wenjing Xu, Qianyu Zhu, Jiaxing Chen.

**Formal analysis:** Jiaxing Chen.

**Investigation:** Aijie Yuan.

**Methodology:** Aijie Yuan, Peng Cao.

**Resources:** Junchen He.

**Software:** Aijie Yuan, Peng Cao.

**Supervision:** Junchen He, Litao Zhang.

**Validation:** Peng Cao, Litao Zhang.

**Visualization:** Wenjing Xu, Jiaxing Chen.

**Writing – original draft:** Wenjing Xu, Jiaxing Chen.

**Writing – review & editing:** Qianyu Zhu, Litao Zhang.
